# Prioritisation and the initiation of HCC surveillance in CHB patients: lessons to learn from the COVID-19 crisis

**DOI:** 10.1136/gutjnl-2020-321627

**Published:** 2020-05-25

**Authors:** Georgia Zeng, Upkar S Gill, Patrick T F Kennedy

**Affiliations:** 1 Faculty of Medicine, UNSW, Sydney, New South Wales, Australia; 2 Barts Liver Centre, Immunobiology, Blizard Institute, Barts and The London School of Medicine & Dentistry, Queen Mary University of London, London, UK

**Keywords:** hepatitis B, hepatocellular carcinoma, surveillance

## Premise

COVID-19 was declared a pandemic by WHO in March 2020 resulting in an unprecedented strain on healthcare systems globally.^1^ Currently, there is no proven treatment for severe acute respiratory syndrome coronavirus 2 (SARS-CoV-2, which can cause serious disease with an associated high mortality in a proportion of patients.^2^ The impact of COVID-19 was first seen in the Chinese healthcare system, but the experiences of Italy, France, Spain, the UK and now the USA underline the gravity of the crisis and the challenge that healthcare professionals will have to overcome globally.^3–5^

The current pandemic has impacted the management of almost all patients and those with chronic liver disease are no exception. These are uncertain times for both patients and healthcare professionals, while we adjust to the threat posed by COVID-19. An area of specific concern for us is how best to provide hepatocellular carcinoma (HCC) surveillance in chronic hepatitis B (CHB) patients and importantly, how we will prioritise patients for HCC screening at a time of limited resources, concern over potential nosocomial transmission and strict social distancing. While new guidance in relation to COVID-19 suggests that HCC surveillance can be deferred,^6 7^ it is accepted that patients with cirrhosis, elevated serum alpha-feto protein (AFP) and those with CHB among others should be prioritised. However, we feel that this may also represent an opportunity to review and streamline the risk stratification of CHB patients in whom, and when, we offer HCC surveillance. In this article, we outline the pertinent clinical aspects regarding CHB and HCC, in addition we provide a review of current surveillance guidance and risk stratification models. We reflect on the impact of COVID-19 infection on HCC surveillance and how this crisis could be used as a springboard to determine the timing of initiation of HCC surveillance, by developing novel risk stratification models for CHB patients in the future.

## Hepatitis B virus

Chronic hepatitis B virus (HBV) infection is the leading cause of HCC worldwide, with 30% of the global population showing serological evidence of current or past infection.^8^ It is estimated to result in 780 000 deaths per year, the vast majority of which are attributable to cirrhosis and HCC; with cirrhosis being the most important risk factor for HCC development.^9 10^ Universal vaccination programmes for HBV prophylaxis have been successfully adopted in many countries, resulting in reduced mortality.^11 12^ Furthermore, potent antiviral therapy has also significantly impacted HBV outcomes and nucleos(t)ide analogue (NA) therapy has been reported to lower HCC incidence.^13 14^ Patients exposed to HBV perinatally or in early childhood are typically characterised by high level HBV DNA in the absence of significant liver injury.^15^ This initial disease phase was previously referred to as ‘immune tolerant’, but concerns have been raised regarding HBV DNA integration and clonal hepatocyte expansion, events associated with hepatocarcinogenesis. A change in the nomenclature in the 2017 European Association for the Study of Liver (EASL) guidelines to ‘e-Antigen positive chronic infection’, underscored the possibility of disease progression and the risk of HCC development during this disease phase.^16^ The emergence of a subsequent immune response with perturbation in liver enzymes and reduction in HBV DNA, leads to the ‘immune clearance’ phase (e-Antigen positive chronic hepatitis).^17 18^ An integral event is hepatitis B e antigen (HBeAg) seroconversion and importantly, patients achieving this before the age of 30 are recognised to have a lower chance of progression to cirrhosis and HCC.^19^ The majority of individuals become ‘inactive carriers’ after spontaneous HBeAg seroconversion, defined as the ‘e-Antigen negative chronic infection’ phase, which is associated with a lower risk of progression to cirrhosis and HCC.^20^ Conversely, reactivation due to the presence of core and pre-core mutants can lead to elevated HBV DNA and alanine aminotransferase (ALT) causing disease progression and HCC development. Thus, disease phase along with other recognised patient factors such as age, ethnicity and family history of HCC govern the timing of HCC surveillance.

## Hepatocellular carcinoma

HCC is the third-leading cause of cancer-related death worldwide with the highest incidence in Africa and Asia.^21^ The majority of HCC cases arise in the context of underlying cirrhosis, with incremental effect of risk factors such as hepatotropic viruses, alcohol misuse and the metabolic syndrome,^22^ but importantly HBV is an oncogenic virus, thus HCC can occur in the absence of advanced liver damage or cirrhosis.

An important component of hepatocellular carcinogenesis is HBV DNA integration, which preferentially occurs at sites of double-stranded breaks in genomic DNA.^23^ HBV X protein is a functionally active component of integrated HBV DNA that promotes cell cycle progression, silences tumour suppressor genes and instigates chromosomal instability.^24^ HBV DNA integration can be used as a marker of expanding hepatocyte lineages, which can promote a HBV resistant state in response to persistent immune killing of infected hepatocytes.^25^ Importantly, we and others have shown that events such as HBV DNA integration and clonal hepatocyte expansion are already present in patients in the early phase of disease,^26 27^ and this is a key tenet to the development of HCC in non-cirrhotic individuals with CHB infection. It is, therefore, critical to consider these molecular dynamics of HBV for HCC surveillance. Separate meta-analyses demonstrate that specific HBV mutations^28^ and genotype C infection^29^ are associated with an increased risk of HCC. Non-modifiable host factors as previously mentioned; older age and male sex; have consistently been validated as independent risk factors for HCC and included in various HCC risk scores, discussed further in this review. Other risk factors for HCC development include excessive alcohol intake,^30^ aflatoxin exposure,^31^ coinfection with hepatitis D virus (HDV), HIV^32 33^ and now increasingly recognised, the metabolic syndrome.^34^ In a 2015 case–control study, evidence of the metabolic syndrome (presence of diabetes, obesity in early adulthood) increased the OR for HCC development by sixfold. Although the OR for the development of HCC in CHB patients is 30–40,^35^ it is not proven whether the presence of CHB in conjunction with the metabolic syndrome leads to a synergistic effect on HCC development. A recent study reported that patients with CHB and liver steatosis had a greater mortality and risk of developing HCC compared with CHB patients without steatosis.^36^ Thus, the metabolic syndrome is emerging as an important coaetiology with CHB when evaluating HCC risk.

## HCC surveillance guidelines

Advances in the understanding and treatment of HBV necessitate regular updating of HCC surveillance guidelines. Evaluation of international guidelines, (table 1), reveal discrepancies between the specified populations at risk. While all guidelines agree that the presence of cirrhosis mandates regular surveillance, they differ in how they broadly stratify the ever changing concept of HCC risk. The EASL guidelines^37^ identify, but fall short of recommending conventional risk scores, each of which is based on a varying combination of host and viral factors. These risk scores are affirmed in the Asian Pacific Association for the Study of the Liver (APASL) guidelines,^38^ aligning with the fact that most are validated in Asian populations, but are omitted from the American Association for the Study of Liver Diseases (AASLD) guidelines.^39^ There is broad agreement, however, across the liver disease organisations on the use of abdominal ultrasound scan (USS) every 6 months as the appropriate method of HCC surveillance. The AASLD and APASL guidelines differ on their recommendation of using serum AFP for risk assessment, as studies have demonstrated the lack of sensitivity and specificity of AFP in diagnosing HCC.^40 41^ Importantly, during the COVID-19 pandemic, there is acceptance in the field that HCC surveillance can be deferred,^6 7^ thus, both healthcare professionals and patients will have to accept delays in access to liver imaging in the short term. However, there is an onus on specialists to provide an exit strategy to this disruption, or put simply, provide a cogent way forward to prioritise those at greatest need for HCC surveillance.

**Table 1:** Comparison of international HBV guidelines for HCC surveillance

	EASL (2017)	AASLD (2018)	APASL (2015)
Who?	Suggests consideration of host and disease factors Suggests risk scores (*eg, PAGE-B*) to stratify patients as low, moderate or high risk Recommends surveillance for patients: Undergoing long-term NA therapy With cirrhosis With moderate/high risk at onset of NA therapy Recommends against surveillance for patients: With low risk	Recommends surveillance for HBsAg+ patients with: Cirrhosis High risk (Asian/Black men *>*40 years, Asian women >50 years) History of HCC in first degree relative HDV coinfection	Affirms risk prediction scores can accurately risk stratify patients Suggests threshold incidence of surveillance be determined individually based on the economic situation of each country Recommends surveillance for patients with: CHB at high risk Recommends against surveillance for patients with: Class C Child-Pugh scores.
How?	Not stated	Recommends ultrasound±AFP 6 monthly States insufficient evidence for/against inclusion of AFP in screening algorithms	Recommends USS+AFP 6 monthly, or preferably 3 monthly in cirrhotics and those at high risk Recommends contrast-CT or contrast-MRI to confirm suspicious ultrasound lesions AND for initial screening of patients with advanced cirrhosis and high suspicion of HCC development Recommends baseline contrast-CT or contrast-MRI obtained in all cirrhotics at presentation

AASLD, American Association for the Study of Liver Diseases; AFP, alpha fetoprotein; APASL, Asian Pacific Association for the Study of the Liver; CHB, chronic hepatitis B; EASL, European Association for the Study of the Liver; HBsAg+, hepatitis B surface antigen positive; HBV, hepatitis B virus; HCC, hepatocellular carcinoma; HDV, hepatitis D virus; NA, nucleos(t)ide analogue.

## HCC risk scores

In the current COVID-19 era of reducing or potentially delaying HCC surveillance, the use of risk scores in CHB patients may enhance stratification, which could be integrated in future practice to prevent unnecessary tests. The early risk stratification scores, including Guide with age, gender, HBV DNA, core promoter mutations and cirrhosis (-HCC,^42^ cumulative HCC (CU-HCC)^43^ and Risk Estimation for Hepatocellular Carcinoma in Chronic Hepatitis B (REACH-B),^44^ consider patient demographics and disease-related factors. All three conventional risk scores include HBV DNA; where a high HBV DNA was thought to directly correlate with HCC development, despite HBV being a non-cytopathic virus.^45^ Conversely, recent data have shown that HBV DNA is not independently associated with HCC occurrence in patients treated with NA therapy,^46 47^ justifying its exclusion in newer risk scores. Indeed, more recent studies suggest the incorporation of HBV DNA may reduce the predictive capacity of these models, as the natural history of CHB has evolved in the era of widespread antiviral uptake.^48^

The discriminatory performance of these three risk scores in Caucasian patients has been limited,^49 50^ possibly due to differences in genotype distribution. In contrast, the PAGE-B score (based on patients’ age, gender and platelets) was modelled around a treated Caucasian cohort,^51^ and has since been validated in treated Asian patients, showing similar or superior predictive performance to the GAG-HCC and CU-HCC risk scores.^52^ A modified PAGE-B (mPAGE-B) score has been developed in a treated Asian cohort, where serum albumin as an independent predictor of HCC development prompted its inclusion as a factor in the risk model.^53^ The mPAGE-B score demonstrated better predictive performance than the original PAGE-B score and other conventional risk models in the original study,^53^ but an external validation study has demonstrated similar performance compared with the original PAGE-B score.^54^

While hepatic fibrosis, and specifically cirrhosis, is the single most important risk factor for HCC,^55^ the invasive nature of liver biopsy has precluded its inclusion in older HCC risk models. However the adoption of transient elastography (TE) allows for accurate and non-invasive means of liver stiffness measurement (LSM). TE has now been included in the development of new risk scores,^56^ as well as the modification of old scoring systems,^57 58^ these contemporary risk scores have been outlined in table 2. While the LSM-Based Model uses LSM as a continuous variable, the LSM-HCC and modified REACH-B (mREACH-B) models use discrete cut-offs to stratify patients into three categories for simpler scoring. The LSM-HCC model modifies the CU-HCC score by replacing USS as a surrogate measure of cirrhosis with LSM, while the mREACH-B score substitutes HBV DNA with LSM. Both of these modified risk scores yield better predictive performance than their original models.^57 58^ In external validation studies, the LSM-HCC and mREACH-B scores demonstrate superior performance in comparison to conventional risk scores, as well as the PAGE-B score, which are best used in patients established on antiviral therapy regardless of HBeAg status.^59 60^ Thus, in the current COVID-19 pandemic where healthcare professionals managing CHB patients are requested to delay standard USS imaging, such modified risk scores could easily be used to better stratify the need for HCC surveillance in patients. Cut-off scores for the PAGE-B model are specified, but this is not the case for the mREACH-B score, however, the validation study for this model did propose thresholds. Utilising these cut-off scores allows for the improved interpretation of these models to determine the timing of HCC surveillance; these are summarised in table 3. The cut-offs provided for both risk models are in accordance with the reported 5-year CU-HCC incidence rates, approximately 0% for low risk, 3%–4% for intermediate risk and >10% for high risk. Thus, using these scores, with their designated thresholds would lead to more robust identification of ‘at-risk’ patients for earlier HCC surveillance, when healthcare services return to standard activity in due course.

**Table 2:** Details of contemporary HCC risk scores

Risk score	Components	Original cohort	Validation (V)/performance (P)
LSM-Based Model (2013)^56^	Age Gender HBV DNA LSM	Asian cohort (mixed treatment status)	V; none in other cohorts
LSM-HCC (2014)^57^	Age Albumin HBV DNA LSM	Asian cohort (mixed treatment status)	Modification of CU-HCC: V: Asian cohorts (mixed treatment status)^59 60^ P: superior to conventional risk scores and PAGE B,^60^ similar to mREACH-B^59 60^
mREACH-B (2014)^58^	Age Gender ALT HBeAg status LSM	Treated Asian cohort	Modification of REACH-B: V: Asian cohorts (mixed treatment status)^48 59 60^ P: superior to conventional risk scores^48 59^and contemporary risk scores^59 60^
PAGE-B (2016)^51^	Age Gender Platelets	Treated Caucasian cohort	V: treated Asian cohorts^52 54 60^ P: similar or superior to conventional risk scores,^52^ similar to mPAGE-B^54 60^ but inferior to LSM-HCC and mREACH-B^60^
mPAGE-B (2018)^53^	Age Gender Platelets Albumin	Treated Asian cohort	Modification of PAGE-B: V: treated Asian cohort^54^ P: similar or superior to conventional risk scores^54^

ALT, alanine aminotransferase; CU-HCC, cumulative HCC; HBeAg, hepatitis B e antigen; HBV, hepatitis B virus; HCC, hepatocellular carcinoma; LSM, liver stiffness measurement; mPAGE-B, modified PAGE-B.

**Table 3:** Interpretation of recommended risk scores

Risk score	Score	Risk interpretation	Recommendation
mREACH-B^58^	≤5	Low risk	HCC surveillance not recommended
	6–7	Low-intermediate risk	HCC surveillance recommended
	8–10	High-intermediate risk	HCC surveillance recommended
	≥11	High risk	HCC surveillance strongly recommended
PAGE-B^51^	≤9	Low risk	HCC surveillance not recommended
	10–17	Medium risk	HCC surveillance recommended
	≥18	High risk	HCC surveillance strongly recommended

HCC, hepatocellular carcinoma.

## Quantitative hepatitis B surface antigen and hepatitis B core-related antigen as new biomarkers for HCC risk

HBV DNA in the context of antiviral therapy is less discriminatory and this has been reflected in the evolution of HCC risk scores. This heralds the potential role of new biomarkers such as quantitative hepatitis B surface antigen (qHBsAg) and hepatitis B core-related antigen (HBcrAg) levels, which may also inform HCC risk. HBsAg levels vary over the natural course of CHB and typically decrease over time, as patients progress to e-Antigen negative chronic infection,^61^ while late-stage elevation of HBsAg levels may reflect the development of e-Antigen negative chronic hepatitis or HBV DNA integration.

The recently published SONIC-B study has pooled data from eight global randomised trials to support the hypothesis that HBsAg levels inversely correlate with severity of fibrosis and cirrhosis in HBeAg positive patients.^62^ This aligns with previous, smaller-scale natural history studies,^63 64^ demonstrating the clinical utility of HBsAg cut-offs to rule out cirrhosis. These findings support the hypothesis that lower serum HBsAg levels imply longer durations of HBV-related hepatic inflammation, and are in keeping with evidence that longer duration of HBsAg exposure leads to attrition of virus-specific T cell responses.^65^

However, it is noteworthy that HCC risk has been shown to be significantly reduced in HBeAg negative patients who achieve HBsAg seroclearance, either spontaneously^66 67^ or with antiviral therapy.^68 69^ Studies have shown that HBsAg levels are independently associated with the development of cirrhosis and HCC in patients with low levels of HBV DNA.^70 71^ A positive correlation between HBsAg level and HCC has also been reported in patients with intermediate (grey-zone) viral load, defined as HBV DNA 2000–20 000 IU/mL,^72^ although the timing of qHBsAg measurement is vital to its interpretation and utility.

While qHBsAg proves an important marker in monitoring disease progression and/or treatment response in patients with low level viraemia, there is a need to validate a safe HBsAg cut-off level as a threshold for HCC surveillance. Taiwanese data demonstrated that higher HBsAg levels correlated with HCC risk particularly in e-Antigen negative patients with HBV DNA <2000 IU/mL. The authors proposed a HBsAg threshold of 1000 IU/mL to delineate HCC risk, with an adjusted HR for HCC development of 13.7 (95% CI 4.8 to 39.3) in their cohort of treatment-naïve patients.^70^ Newly proposed algorithms have used this threshold to complement HCC risk stratification in e-Antigen negative patients with HBV DNA <2000 IU/mL and normal ALT levels.^73^ Additionally, the incorporation of HBsAg levels into a HCC risk score modelled off data from the REVEAL-HBV study has demonstrated exceptional prediction accuracy and discriminatory ability for 5-year, 10-year and 15-year HCC risk. In this analysis, CHB patients demonstrated multivariate-adjusted HRs for HCC development of 2.83 (1.55–5.18) and 4.06 (2.24–7.36) for patient groups with HBsAg levels 100–900 and >1000 IU/mL respectively, in comparison to those with HBsAg level <100 IU/mL.^71^

HBcrAg, combining antigenic reactivity resulting from HBcAg, denatured HBeAg and artificial core-related protein p22cr has been found to correlate well with serum HBV DNA until the initiation of antiviral therapy, at which point HBcrAg declines at a slower rate than HBV DNA.^74^ As such, it can be regarded as a marker for persistence of HBV, and has been found to significantly correlate with intrahepatic cccDNA in liver biopsy studies.^75–77^ HBcrAg levels have been found to independently predict hepatocarcinogenesis in untreated patients,^78 79^ as well as patients receiving NAs.^80–82^ In fact, it may be superior to HBsAg in predicting HCC risk in treated patients with undetectable HBV DNA,^83^ underlining its potential utility in the future.

To our knowledge, there are no published risk models that incorporate HBcrAg levels, nor is there a consensus on a safe HBcrAg cut-off level to help inform the decision of whether to initiate HCC surveillance. Correspondingly, any existing or upcoming risk models that incorporate HBcrAg levels require validation in cohorts of patients with different demographics including gender, age, ethnicity, viral genotype and treatment status. The design of future studies should align with these goals in order to increase the relevance of HBcrAg and qHBsAg for practising clinicians. Indeed, viral and host factors, such as metabolic syndrome, alcohol intake and coinfection, are not included in current risk scores, but should be part of future risk stratification. Importantly, future research will have to address the utility of novel viral markers, molecular events associated with HBV DNA integration and host factors in determining HCC risk prior to their incorporation and validation in new models. We have outlined these various factors, which could be used to improve HCC risk scores in the future (table 4). The advent of novel risk scores and models incorporating these parameters, along with those already included in validated scores, may further improve risk stratification for HCC surveillance, which could be adopted to determine the optimal timing for the initiation of HCC surveillance in a personalised way in the future.

**Table 4:** Coaetiologies, molecular events and novel parameters for individual HCC risk assessment

Future research directions in assessing HCC risk	Examples of parameters
Incorporating new viral factors	Specific HBV mutations
	HBV genotype*
	HBV DNA integration
Incorporating new host factors	Metabolic syndrome
	Excessive alcohol intake
	HDV or HIV coinfection
	Other hepatic co-aetiologies
	Treatment status†
Incorporating new quantitative biomarkers	qHBsAg
	HBcrAg

*Inclusion of all HBV genotypes.

†Inclusion of patients regardless of treatment (naïve and experienced patients).

HBcrAg, hepatitis B core-related antigen; HBV, hepatitis B virus; HCC, hepatocellular carcinoma; HDV, hepatitis D virus; qHBsAg, quantitative hepatitis B surface antigen.

## A way forward

### Prioritisation during COVID-19

The COVID-19 crisis has brought great disruption to healthcare systems across the world, significantly impacting established care pathways and planned patient activity, in order to meet the challenge of the global pandemic. Regrettably, HCC surveillance programmes along with many other cancer screening programmes have been and will continue to be disrupted as a consequence of this public health emergency for some time. In these challenging times, it is imperative that we adapt clinical practice in a timely way to maintain the highest standards of patient care and safety. During the COVID-19 crisis, we propose a strategy to streamline HCC surveillance, a preventative programme, which is critical to the long-term health of at-risk CHB patients. In light of the current urgency, we suggest that clinicians commit to using contemporary HCC risk scores to inform the risk stratification of their CHB patients. We recommend the mREACH-B score as the preferred risk model for CHB patients currently taking or maintained on antiviral therapy. In the absence of the availability of a LSM reading, which is likely to be the case in less economically developed countries, we recommend the PAGE-B score. Using the cut-off scores, as outlined in table 3, patients who require more urgent HCC surveillance can be identified and prioritised.

### Development of future risk scores

Moving forward clinicians and healthcare workers involved in the care of CHB patients should learn the ‘lessons’ from the COVID-19 crisis to improve future HCC surveillance. Initially, by adopting the contemporary HCC risk models with their established cut-off scores for prioritisation, the timing and initiation of HCC surveillance could be enhanced. We envisage that HCC risk scores will be improved further by the inclusion of the additional factors outlined in table 4. Future research will have to evaluate the effect of host risk factors in HCC development; including the presence of the metabolic syndrome, alcohol use and coinfection; which are currently not accounted for in the development of risk scores. Additionally, levels of HBsAg and HBcrAg may be of value in assessing both e-Antigen positive and negative patients, with and without antiviral therapy, and should be incorporated into future HCC risk scores. Critically, it is vital that existing and future risk scores are further confirmed by robust external validation studies in diverse CHB patient cohorts. While these risk models will be of particular value in the midst of the current pandemic, we believe that they can play an important role to inform the decision and timing to initiate HCC surveillance when healthcare systems eventually adapt to a post-COVID era.
